# Assessment of cough in head and neck cancer patients at risk for dysphagia—An overview

**DOI:** 10.1002/cnr2.1395

**Published:** 2021-05-01

**Authors:** Sofiana Mootassim‐Billah, Gwen Van Nuffelen, Jean Schoentgen, Marc De Bodt, Tatiana Dragan, Antoine Digonnet, Nicolas Roper, Dirk Van Gestel

**Affiliations:** ^1^ Department of Radiation Oncology, Speech Therapy, Institut Jules Bordet Université Libre de Bruxelles Brussels Belgium; ^2^ Department of Otolaryngology and Head and Neck Surgery, University Rehabilitation Center for Communication Disorders Antwerp University Hospital Antwerp Belgium; ^3^ Department of Translational Neurosciences, Faculty of Medicine and Health Sciences University of Antwerp Antwerp Belgium; ^4^ Department of Logopaedics and Audiological Sciences, Faculty of Medicine and Health Sciences University of Ghent Ghent Belgium; ^5^ BEAMS (Bio‐, Electro‐ And Mechanical Systems) Université Libre de Bruxelles Brussels Belgium; ^6^ Department of Radiation Oncology, Head and Neck Unit, Institut Jules Bordet Université Libre de Bruxelles Brussels Belgium; ^7^ Department of Surgical Oncology, Head and Neck Surgery Unit, Institut Jules Bordet Université Libre de Bruxelles Brussels Belgium; ^8^ Department of Oto‐Rhino‐Laryngology and Head & Neck Surgery, Erasme Hospital Université Libre de Bruxelles Brussels Belgium

**Keywords:** aspiration, assessment, cough, dysphagia, head and neck cancer, radiation

## Abstract

**Background:**

This literature review explores the terminology, the neurophysiology, and the assessment of cough in general, in the framework of dysphagia and regarding head and neck cancer patients at risk for dysphagia. In the dysphagic population, cough is currently assessed perceptually during a clinical swallowing evaluation or aerodynamically.

**Recent findings:**

Recent findings have shown intra and inter‐rater disagreements regarding perceptual scoring of cough. Also, aerodynamic measurements are impractical in a routine bedside assessment. Coughing, however, is considered to be a clinically relevant sign of aspiration and dysphagia in head and cancer patients treated with concurrent chemoradiotherapy.

**Conclusion:**

This article surveys the literature regarding the established cough assessment and stresses the need to implement innovative methods for assessing cough in head and neck cancer patients treated with concurrent chemoradiotherapy at risk for dysphagia.

**Abbreviations**
C2 (C5): the dose of tussive agent required to elicit two (five) coughs
CCRT: concurrent chemoradiotherapy
CSE: clinical swallow examination
FEES: fiberoptic endoscopic evaluation of swallowing
HNC: head and neck cancer
RAD: late radiation‐associated dysphagia
RT: radiotherapy
TRP: transient receptor potentials
VFSS: Videofluoroscopic Swallow Study

## 1 INTRODUCTION

Late radiation‐associated dysphagia (RAD) can be defined as impaired swallowing safety and/or efficiency following intensive non‐surgical treatment regimens in head and neck cancer (HNC) patients.^1^, ^2^ It has been reported by 50 to 79% of HNC‐patients treated with concurrent chemoradiotherapy and has a high impact on patients' reported quality of life.^4^, ^5^, ^6^, ^7^

The two hallmarks of RAD are residue (ie, food sticking in the oral cavity, pharynx, or larynx), and penetration/aspiration.^9^, ^10^ Penetration is defined as an entry of material into the larynx, but which does not pass below the vocal folds. Aspiration is defined as an entry of material below the vocal folds.^11^ In case the patient's sensory system is unimpaired, penetration and aspiration result in a cough reflex that protects the lower airways and lungs by evacuating the material.^11^ In HNC‐patients, however, the efficacy of the elicited cough is often diminished or absent due to sensory deterioration.^12^

Radiation‐induced deterioration is characterized by inflammatory changes, which may result in enhanced cough response (higher sensitivity/reactivity due to inflammation) in the early phase. Because of cell depletion, inflammation (ie, heat, pain, swelling), xerostomia, dysgueusia, mucositis and desquamation may occur.^13^, ^14^ Those acute troubles generally appear within 3 months and resolve within a few months.^13^ In the late phase, delayed radiation injuries may depend on the radiation dose or be the consequence of acute troubles^14^, ^15^ with an exponential decay 12 to 18 months after treatment.^14^

Late radiation injuries involve fibrotic lesions and muscle atrophy. This may alter nerve electrophysiology and lead to hyposensitivity. Sensory deterioration in this head and neck region is a serious threat as it may result in an inefficient cough reflex.^14^, ^16^, ^17^, ^18^, ^19^ Therefore, HNC‐patients with RAD are severely at risk of food aspiration.

Literature has reported high incidence rates of aspiration in this population ranging between 20% and 83%.^6^, ^20^, ^21^, ^22^, ^23^, ^24^, ^25^, ^26^, ^27^, ^28^, ^29^, ^30^, ^31^, ^32^, ^33^, ^34^ It has been observed that aspiration rates depend on the adjunct of other treatments such as chemotherapy or surgery.^24^, ^32^, ^34^ Jagtap et al reported a higher risk of penetration/aspiration after combining a tongue base resection with radiotherapy. Moreover, 50% of the patients treated with radiotherapy (RT) presented silent aspirations.^32^ Patients treated with concurrent chemoradiotherapy (CCRT) for oral, oropharyngeal, nasopharyngeal and laryngeal cancers have exhibited rates of aspirations as high as 83%.^20^ Following RT alone, aspiration occurs in up to 48% of the patients treated for oral, oropharyngeal, and laryngeal cancer^22^, ^23^ and in up to 65.9% of the patients treated for nasopharyngeal cancer.^33^

Hedström et al have developed the DESdC, a study‐specific categorical symptom score to determine patient‐reported dysphagia (DESdC = presence of Drinking, Eating, Swallowing difficulties and Coughing when eating/drinking [any combination]; scores between 0 and 4 with 0 = no symptom).^35^ Their study has reported that 89% of HNC‐patients treated with RT or CCRT reported symptoms of RAD. The most commonly reported DESdC score was 3 (33%). Because of the occurrence of silent aspirations, fewer patients (13%) reported four symptoms (including coughing). According to the Penetration‐Aspiration scale,^11^ these patients were severely at risk of aspiration‐pneumonia but they were not aware of it. Besides, Rogus‐Pulia et al demonstrated that 83% of aspirations and 100% of penetrations in patients treated with CCRT are silent.^20^

Mortensen et al reported dysphagia‐related aspiration and aspiration pneumonia to remain the most life‐threatening complications of radiotherapy in HNC‐patients.^34^ The mortality rate in patients with RAD developing aspiration pneumonia after CCRT ranges from 9% to 34.6%.^21^, ^24^, ^25^ The risk of aspiration pneumonia in RAD patients may occur up to 10 years after treatment.^6^, ^25^, ^26^, ^36^ Early detection of dysphagia and especially aspiration is therefore key in this population.

## 2 ESTABLISHED DYSPHAGIA ASSESSMENT

Videofluoroscopic Swallow Study (VFSS) is considered the gold standard for evaluating dysphagia.^37^, ^38^, ^39^ The direct and continuous visualization of the oral, pharyngeal, and cervical esophageal phase of swallowing, allowing for symptom detection and simultaneously revealing crucial information regarding swallowing physiology, is a major advantage of this instrumental, radiographic examination.^3^, ^20^ As an adjunct to VFSS, the Fiberoptic Endoscopic Evaluation of Swallowing (FEES) is a useful tool in providing direct imaging of superior pharyngeal anatomy, secretions, and vocal fold movement.^3^ As each tool has its own strengths and plays a complementary role with regard to the other, combining the two improves their sensitivity for detecting aspiration and residue.^40^

Due to lack of availability, patient compliance, and expertise, it is not possible to carry out instrumental examination in daily clinical practice on every patient suspected of dysphagia.^41^ Therefore, evaluation of swallowing efficacy and safety often starts with a “bedside” or clinical swallow examination (CSE). The CSE comprises multiple liquid and food swallowing trials,^3^ patient history, assessment of the oral mechanism, and patient‐reported outcome. To diagnose dysphagia and aspiration, clinicians often rely on identifiers of dysphagia and aspiration risk like abnormal coughing, dysphonia, dysarthria, abnormal gag reflex, and post‐swallowing voice change.

Previous research has shown that CSE underestimates aspiration risk in patients at risk and overestimates aspiration risk in patients without risk.^6^, ^9^, ^34^, ^42^, ^43^ This is possibly due to the clinical markers being unrelated to swallowing (eg, gag reflex) or unreliable indicators of penetration and aspiration (eg, voice quality).^42^, ^44^, ^45^, ^46^, ^47^, ^48^ Despite limitations in estimating swallowing safety accurately, the CSE represents the more accessible method for assessing dysphagia in daily clinical practice.

## 3 COUGH TERMINOLOGY AND COUGH NEUROPHYSIOLOGY

Prior to exploring established cough assessment, cough definition, and cough type categorization, as well as a description of the neurophysiology of coughing, are warranted.

### 3.1 Terminology and types of coughing

Coughing is defined as a deep inspiration followed by closure of the glottis (compression), forced expiratory effort, and then opening of the glottis with expiration.^12^ As mentioned, effective coughing plays a crucial role in expectorating foreign bodies from the airways and avoiding aspiration, particularly during swallowing in patients with RAD.^49^

Differences exist with regard to cough sequencing. A single cough is one cough produced after one inspiration. A cough epoch or cough bout, or cough attack as commonly named by patients, represents successive coughs after one single inspiration.^43^, ^50^, ^51^ By multiple coughs, one designates successive coughs separated from each other by an inspiration.

Coughing may be either voluntary or reflexive. A voluntary cough originates in the cerebral cortex. Voluntary cough testing involves asking a patient to cough. A reflexive cough is elicited by direct activation of receptors on airway sensory nerves.^52^ The reflexive cough is induced by stimuli that motivate the subject to protect and clear the airway by coughing^53^ and is always preceded by an urge‐to‐cough, a biological perceived need to cough.^53^

Whereas a (reflexive or voluntary) cough starts with an inspiratory phase, the expiratory reflex following contact of food, liquids, or chemicals with the true vocal folds or the upper tracheal areas, starts with a closure of the glottis without any prior inspiration.^51^ This is possible because swallowing (food or liquids) physiologically starts during the expiratory phase and interrupts quiet breathing. The lung volume during quiet breathing is ranging from 42% to 48% of total vital capacity.^54^ This lung volume is sufficient to allow the expiratory reflex to occur during swallowing without prior additional inspiration.^54^ During laryngeal penetration or aspiration, it is the expiratory reflex that is required rather than reflexive cough because the latter starts with an inspiration that would pull foreign material deeper into the lungs. Despite a difference in kind between the two reflexes, this dissimilarity is not always taken into account in the framework of the management of dysphagia.^54^

Unlike coughing that requires complete glottal closure, throat clearing is another reflexive or voluntary maneuver that removes laryngeal residue or mucus, but which involves partial vocal fold adduction.^43^ Like the expiratory reflex, throat clearing does not have an inspiratory phase.^49^

### 3.2 Neurophysiology of cough

The purpose of this section is to highlight the key role of the stimulated receptors conducive to a cough reflex and the cortical involvement in voluntary coughs. Hereafter, the term cough reflex is used as a synonym of expiratory reflex.

#### 3.2.1 Afferent nerves

Airway sensory nerves activation constitutes the first stage of the cough reflex. Via animal models (mainly guinea pigs), some studies have inferred that human airway afferent nerves may originate from the vagal nodose (inferior) and jugular (superior) ganglia.^44^ Despite heterogeneity, primary afferent cough fibers have been consensually divided in two main groups according to their responsiveness to stimuli, that is, chemically and mechanically stimulated nociceptors.^39^

Chemical receptors are characterized by the presence of Transient Receptor Potential Channels (TRP). TRPs are ion channels found on airway sensory nerves in the trachea, bronchi, and nasal mucosa. Chemical receptors are commonly associated with C‐Fibers, located in the jugular superior ganglia. C‐Fibers are unmyelinated, which explains their chemical responsiveness.^49^ C‐Fibers can sensitize but do not properly trigger cough.^49^ Chemoreceptors are sensitive to mediators present during inflammation, irritation, and modifications in pH.^39^ In contrast, they are relatively insensitive to mechanical stimuli because they involve 100 times higher thresholds than mechanoreceptors.

The cough receptors, described by Widdicombe in 1954, are myelinated and respond to mechanical stimulators such as food and liquids. Therefore, cough receptors play a key role regarding the detection of penetration and aspiration. They are found in the extra‐pulmonary airways (larynx, trachea, mainstem bronchi).^55^ Mechanoreceptors are insensitive to direct chemical stimulation.

The pulmonary stretch receptors are also activated mechanically. They innervate the intrapulmonary airways and are responsible for the duration and magnitude of the inspiratory and expiratory phases during coughing. Consequently, stretch receptors help in regulating the respiratory rate but their concrete role in cough remains uncertain.^56^

The depolarization of chemical and mechanical nociceptors following stimulation leads to an action potential that transmits along the vagus nerve to the nucleus tractus solitaries where central and peripheral responses involving the nucleus ambiguous, the retroambigualis, and the phrenic nucleus are processed.

#### 3.2.2 Cortical control

The cerebral cortex plays an important role in the cognitive processes participating in the modulation of voluntary coughing or voluntary suppression of the cough reflex.^57^ The involved cortical areas are the motor cortex, the sensory motor cortex, the supplementary motor area, and the limbic system. The magnitude of the cortical activation depends on the area of the cerebral cortex.^57^ Voluntary coughs encode the premotor and motor cortex, cerebellum and corticospinal pathway.^56^ Voluntary coughs occur without medullary input. The urge‐to‐cough involves the primary sensory cortex (intensity of the urge‐to‐cough), insula (magnitude of the input from the airway), and the prefrontal and post‐parietal areas (attention and localization of the site of irritation).^56^ Voluntary cough suppression encodes different areas such as anterior insula, supplementary motor area, motor cingulate cortex and right inferior frontal gyrus.^56^, ^58^ The limbic brain contributes to affective factors such as unpleasantness associated with airway irritations and cough.^56^

#### 3.2.3 Efferent nerves

Vagus, phrenic, and spinal motor nerves are activated after motor information processing in the cerebral cortex and cerebellum.^49^, ^59^ These activations cause diaphragm relaxation, thoracic, and abdominal muscle contraction (expiratory and accessory muscles).^49^, ^59^ The nucleus retroambigualis, via phrenic and other spinal nerves, activates inspiratory and expiratory muscles. The nucleus ambiguus, via the recurrent laryngeal nerve (branch of the vagus), sends impulses to the larynx that cause glottal closure.^59^, ^60^ Expiratory and accessory muscle activations differ between a voluntary and a reflexive cough.^60^ A voluntary cough involves a sequential and coordinated activation starting with the expiratory muscles. Accessory muscles respond increasingly afterwards. In contrast, for reflexive coughs, expiratory and accessory muscles are activated simultaneously. That difference suggests that the level of activation in voluntary coughs can be modulated depending on the need perceived.^60^

## 4 ESTABLISHED COUGH ASSESSMENT

Established cough assessment currently comprises subjective ratings and recordings of aerodynamic and acoustic features. With regard to the main topic of this overview, various causes and diagnoses of coughing are not addressed.

### 4.1 Subjective ratings

Persistent coughing is a common unpleasant reason for seeking medical care.^45^ Respiratory diseases like chronic cough or chronic obstructive pulmonary diseases are generally addressed by questionnaire completions, patient history interviews, symptom ratings, clinical observations, and non‐instrumental assessment (eg, ordinal scores or visual analogue scales of subjective cough severity).^61^, ^62^

### 4.2 Aerodynamic features

When a more thorough examination is warranted, patients are subjected to an instrumental evaluation involving pulmonary function testing.^49^ To date, voluntary cough testing is usually carried out by recording the airflow rate using a facemask or pipe coupled to a filter and connected to a digital spirometer.^43^, ^50^ Participants are asked to take in a maximal breath and then produce a volitional cough in the spirometer. They are typically instructed to “cough as hard as they can” or “like they have something stuck in their throat.”^3^

Cough reflex sensitivity testing in general informs on hypersensitivity of the upper or lower airways and the ventilatory capacity.^49^ Cough reflex sensitivity testing involves an inhalation challenge by known tussive agents. Chemoreceptors described above are sensitive to chemicals such as capsaicin (chili pepper), bradykinin, or other classical nociceptor stimulants.^12^, ^63^ In contrast, mechanoreceptors are sensitive to mechanical stimulation and acids such as citric acid, low‐chloride solutions, and distilled water.^12^ Citric acid, aerosolized water (fog), and capsaicin are tussive agents widely used in cough testing and can induce cough in a dose‐dependent and reproducible manner.^46^ Typically, three thresholds are relevant during cough reflex sensitivity testing: (1) cough threshold, meaning the lowest dose (eliciting dose) of tussive agent required to induce a single cough^53^, ^64^ (2) C2: the dose of tussive agent required to elicit two coughs (3) C5: the dose of tussive agent used to produce five successive coughs.^46^

During sensitivity testing, participants can be instructed either to cough if they need to – that is, the “urge‐to‐cough” method—or to suppress/inhibit the (urge to) cough as much as possible—the suppressed reflexive cough method.^49^ It was reported that these two methods help to differentiate between the thresholds for natural and suppressed reflexive coughs. The latter informs on the dose at which suppressing/inhibiting a cough is not possible anymore. According to Monroe et al, the natural reflexive cough threshold might be influenced by cortical expectation of cough occurrence during sensitivity testing.^65^ Therefore, the suppressed reflexive cough threshold should be explored because it represents the point where participants can no longer voluntarily control their cough response.^65^ However, Mills et al have demonstrated that healthy subjects may fail to produce suppressed reflexive cough responses (ie, they do not cough) regardless of the dose of citric acid.^66^ This suggests that this task also involves cortical control participation and higher inhibitor processes.^58^ To enable a more precise examination of cough reflex sensitivity testing, some studies have combined both approaches.^67^

Voluntary cough testing and cough reflex sensitivity testing are assumed to provide complementary information.^43^, ^50^, ^64^ Inconsistent measures between voluntary coughs and suppressed reflexive coughs have been reported in healthy volunteers.^64^ Examining peak flows rates and areas under the curve for pressure, voluntary coughs turned out to be stronger than suppressed reflexive coughs because of cortical inhibition of the latter.^64^ Furthermore, the peak expiratory flow rate and the total expired volume have been reported to be features that discriminate between voluntary coughs and natural reflexive coughs (triggered via the urge‐to‐cough method).^68^ The feature values are larger for voluntary compared to suppressed reflexive coughs.^68^ It has been reported that the amount of air inspired prior to coughing (which is larger for voluntary coughs) may significantly influence cough flow rates.^69^, ^70^

Moreover, some studies that investigated objective aerodynamic cough‐related features in airway diseases showed inconsistencies between aerodynamic measurements and patients' subjective complaints (cough ratings and cough‐related quality of life scores mostly).^71^, ^72^, ^73^

### 4.3 Acoustic features

Cough sound detection has been described as a new challenge to assess respiratory diseases both in humans and animals.^74^, ^75^ The literature suggests that each acoustic cough emission is a sequence that begins with a burst/release, followed by a “fricated” fragment (turbulence noise) and a “voiced” fragment.^76^, ^77^ These expected phases enable to decide whether a cough episode has occurred.^76^ Hence, automated acoustic signal monitoring has been proposed to detect cough episodes successfully.^76^, ^78^, ^79^, ^80^, ^81^ Amplitude, frequency, duration, severity (coughs per epoch), and pattern of the cough, for instance, have been considered to be relevant cough features for automatic early detection of respiratory diseases.^75^, ^78^, ^79^, ^80^, ^81^, ^82^, ^83^

The literature has reported few parallels between automatic cough sound analysis and auditory perception.^84^, ^85^ While comparing auditory assessment and automatic categorization, strong correlations were found regarding the distinction between productive (audible mucus noise) and unproductive coughs (inaudible mucus noise).^84^ In accordance with perceptual classification, a doctoral thesis has also reported the successful automatic distinction by means of acoustic features between wet (presence of mucus) and dry (ticklish, irritation) coughs.^80^ The gender might also be identified perceptually via cough sounds.^84^

However, some cough sound features, which have been reliably reported via automatic cough sound analysis, relate inconsistently to perceptual ratings.^43^, ^86^ Laciuga et al have reported perceptual difficulties in differentiating a cough event from a throat clearing, but also from perceptually differentiating a single cough from a cough epoch.^43^ Cough duration may also be auditorily misjudged.^43^ Low agreement between raters with regard to the cough sound strength and the quality (eg, effortful, breathy, strained) was also reported.^43^, ^86^ Furthermore, both the automatic and perceptual identification of a respiratory disease based on cough signals is very poor.^84^, ^85^ These findings highlight the difficulty of interpreting automatic cough signal detection and categorization in terms of perceived timbre as well as the underlying disease.

## 5 COUGH‐RELATED FEATURES AS BIOMARKERS OF LATE RAD IN HNC‐PATIENTS

In this section, methods are discussed for assessing coughing and quantifying cough signals in the framework of the evaluation of swallowing in HNC‐patients with RAD.

### 5.1 Perceptual ratings

Abnormal coughing before, during, or after swallowing is a relevant clinical marker of dysphagia in head and neck cancer.^8^, ^87^ Coughing is generally assessed perceptually during a clinical swallowing evaluation. In a retrospective study with 89 patients who aspirated following radiotherapy for HNC, the cough reflex efficacy was graded by two or three speech therapists. Results showed that the cough reflex was frequently ineffective, intermittently ineffective, or absent several months following treatment (median = 10 months).^8^

The high incidence of silent aspiration in this population makes the occurrence (or absence) of coughing before, during, or after swallowing a weak predictor of penetration/aspiration.^8^, ^34^ In addition, clinicians such as speech therapists, otorhinolaryngologists, and neurologists do not demonstrate strong inter‐rater agreement for cough reflex efficacy scoring.^43^ Laciuga et al explain the low agreement by a lack of expertise in cough physiology prior to the perceptual assessment.^43^ Due to proven weak inter‐ and intra‐rater reliability, the auditory assessment of cough alone must be considered to be an unreliable predictor of aspiration.^88^

As an adjunct to coughing, voice quality is usually assessed immediately following deglutition.^48^ Changes in voice quality are expected to inform about the possible accumulation of saliva or food at the vocal fold level.^89^ Indeed, it has been reported that a change in voice quality may indicate laryngeal dysfunction or the presence of an intruding object at the laryngeal level.^90^ Waito et al (2010) confirm that a normophonic voice after swallowing reflects a lack of aspiration‐penetration.^48^ However, they have not corroborated that a strong correlation exists between aspiration and changes in perceived voice quality (eg, wet voice).^48^ Even though a cough reflex or wet voice are unreliable markers of abnormal swallows individually, McCullough et al found that their combination may represent a reliable method for detecting penetration and aspiration perceptually.^88^

Because cough‐related information is considered to be clinically relevant in patients with RAD, but the reliability of the auditory assessment of cough is considered to be insufficient, researchers and clinicians have investigated the added value of objective examinations.

### 5.2 Aerodynamic features

In the field of dysphagia, cough airflow‐related measures are regarded to be useful physiologic metrics to track airway defense capabilities in at‐risk individuals.^3^, ^91^ Airflow‐related measurements are generally obtained from reflex as well as voluntary coughs. Voluntary coughs are produced upon verbal command, isolated from swallowing. Reflex cough measurements are obtained by means of an inhalation cough challenge according to the guidelines in Section 4.2. Cough airflow‐related features used in the framework of dysphagia include^43^, ^50^, ^92^: (i) the total number of coughs; (ii) the inspiration phase duration in seconds (s); (iii) the total expired volume (TEV) in the cough epoch in liters (L); (iv) the total cough epoch duration in seconds (s), that is, the time interval from the beginning of the expiratory phase of the first cough to the last cough; (v) the peak expiratory flow rate (PEFR) in liters per second (L/s), that is, the peak airflow rate in the expiratory phase; (vi) the peak expiratory flow rise time (PEFRT) in seconds (s); that is, the duration from the beginning of expiration to the largest flow rate peak of the expiratory phase; (vii) the cough volume acceleration in liters per second squared (L/s^2^), that is, the ratio PEFR/PEFRT; (viii) the compression phase duration (CPD) in seconds (s), that is, the time interval from the end of the inspiratory phase to the start of the expulsive phase (glottal closure).

Previous studies have found a relation between aerodynamic measurements during voluntary coughs and the detection via VFSS or FEES of a risk of penetration/aspiration.^93^, ^94^ A study investigating voluntary cough in an amyotrophic lateral sclerosis (ALS) population found impairments in inspiratory and expiratory cough airflow measurements underlying inadequate airway clearance and secretion management.^92^ Moreover, some research has demonstrated the efficacy of voluntary cough airflow features at identifying stroke patients or Parkinson's disease patients at risk for aspiration.^93^, ^94^, ^95^ However, a majority of studies focusing on voluntary cough airflow waveforms or rates could not distinguish between audible aspiration, that is, aspiration followed by an audible cough and silent aspiration (aspiration without any cough response).^96^

Recent research supports cough reflex sensitivity as a biomarker for silent aspiration.^50^ In 2016, Troche et al investigated the relation between cough reflex, voluntary cough airflow measurements and the risk of penetration/aspiration in a Parkinson's disease population (PD) The capsaicin inhalation challenge showed that the cough reflex threshold (C2) discriminates better than voluntary cough between the risk of penetration and the risk of aspiration. Moreover, Hegland et al demonstrated that the peak expiratory flow rate and the total expired volume of the cough reflex are reliable markers of cough effectiveness in PD.^68^ In contrast, the same markers collected with voluntary coughs are overestimated. The capsaicin cough challenge highlighted the critical role of the cough reflex in preventing aspiration. Besides the capsaicin cough challenge, Miles et al reported that low concentrations of citric acid also enable obtaining aerodynamic features predicting silent aspiration.^96^ However, Morice et al observed large inter‐individual variability in aerodynamic cough responses to irritants in the healthy population and highlighted the importance of focusing on intra‐individual changes rather than differences between healthy subjects and patients.^46^

Regarding head and neck cancer patients with RAD, objective voluntary and reflexive cough measurements are still lacking, although a relation has been found between silent aspirations and an ineffective cough reflex.^8^, ^30^, ^34^ Moreover, the use of aerodynamic equipment is not widespread in clinical practice and speech therapists may have an uneven understanding of pulmonary function testing.^49^ In addition, aerodynamic equipment interferes with an evaluation in a natural setting (eg, during a meal).

### 5.3 Possible added value of acoustic cough‐related features in HNC‐patients with late radiation‐associated dysphagia

As reported, cough sound analysis is regarded as a growing line of research, especially with a view to assessing respiratory diseases. However, in the framework of dysphagia, acoustic analysis in general is an underresearched topic. Some studies have investigated the added value of cervical auscultation (CA) in assessing dysphagia.^97^, ^98^, ^99^, ^100^, ^101^, ^102^ CA consists in listening to swallowing sounds with stethoscopes, microphones, or accelerometers in a patient's natural environment.^100^, ^103^, ^104^, ^105^ Due to its inaccuracy, research has demonstrated the risk of using CA as a stand‐alone tool for assessing dysphagia.^97^, ^101^, ^103^, ^104^, ^106^, ^107^, ^108^, ^109^

Comparing healthy swallows and penetration‐aspiration swallows with a tri‐axial accelerometer, Sejdic et al showed that each axis (three anatomical directions) reported distinct information.^97^ This finding suggests that the position and orientation of a sensor influence the recorded signal. Movahedi et al have demonstrated that recordings with distinct transducers (microphones and accelerometers) are idiosyncratic and non‐interchangeable.^106^ Another issue in CA is the identification/interpretation of acoustic components of swallowing because the sensor is placed on a site that moves and produces extra‐sounds.^108^, ^110^ Moreover, CA demonstrates only fair agreement between raters^103^, ^108^ and over‐reports post‐swallowing aspiration.^108^ These findings highlight the need for further investigation to establish CA as a reliable tool before using it for RAD.

Given the importance of effective coughing in patients with RAD and considering the reliability of acoustic cough‐related features found as biomarkers of respiratory diseases,^75^, ^78^, ^79^, ^80^ the exploration of cough sound analysis in RAD may be relevant. However, this topic is under‐researched and no conclusive results have been reported neither in patients with RAD nor in dysphagia in general.^64^, ^111^, ^112^, ^113^ Mills et al have investigated three measurements of the strength of voluntary and suppressed reflexive coughs in healthy individuals.^64^ The measurements included airflow rate, acoustic signal, and air pressure. Based on the analysis of the peak and the area under the curve of each type of recording, the study emphasized the importance of assessing the strength of reflexive coughs rather than voluntary coughs in the dysphagic population. Voluntary and suppressed reflexive coughs showed low correlations. Also, the conclusions have been based on measures of air pressure and flow rate rather than on acoustic cough signals whose features were less accurate and relevant. Umayahara et al have developed a model aiming at predicting the cough peak airflow rate from the cough peak sound pressure level.^111^, ^112^, ^113^ This study showed that the relation between cough airflow rate and cough sounds is affected by microphone distance, patient age, and height but not by weight.^112^ These parameters may modify cough sounds and should be compensated for by a proportionality coefficient in order to control their effect on the cough peak airflow rate estimation. Other factors such as human posture during measurements might also have influenced the model. Further analysis is needed to improve the accuracy and the reliability of cough peak flow measurements based on cough sounds.^111^

To our knowledge, acoustic cough‐related features in HNC‐patients with RAD have not been explored yet. As mentioned previously, inadequate coughing is a clinical cause of aspiration in HNC‐patients with RAD^8^, ^87^ that needs close monitoring by dysphagia experts. Developing an easy, inexpensive, and repeatable method for testing cough in the framework of dysphagia in daily life would offer an alternative method for tracking swallowing problems in HNC‐patients. Such a method would enable exploiting acoustic features related to voluntary and reflex cough as biomarkers of dysphagia and/or aspiration. Recording acoustic cough‐related features is easily implementable in clinical practice and could provide valid measurements. In particular, since cough signals are poor in head and neck cancer patients, such a method could provide novel information regarding this population. Also, given that cough features in isolation are not considered to report reliably on the swallowing function per se,^88^, ^96^ considering other acoustic features describing voice quality could provide additional information for assessing dysphagia amongst head and neck cancer patients with RAD.

## 6 LIMITATIONS AND FUTURE DIRECTIONS

Although there is an increasing interest in cough testing in the field of dysphagia, cough investigation is minimal in head and neck cancer patients with RAD. Cough terminology and typing applicable in a RAD framework are lacking. Perceptual ratings of cough efficiency during a clinical swallowing evaluation show low rater agreement. Regarding objective measures, the literature mainly focuses on the assessment of voluntary coughs to predict the risk of aspiration. However, HNC‐patients treated with CCRT are at risk of silent aspiration. Silent aspirations in this population may occur in up to 83% of the patients and may lead to death in up to one‐third of them. Aspirations cannot be reliably predicted by voluntary coughs. Furthermore, the gold standard for assessing coughing objectively is the instrumental recording of airflow data via a facemask or pipe connected to a digital spirometer. Airflow rate recordings require a piece of equipment that is impractical for routine bedside clinical assessment. This impracticality interferes with daily life assessment, that is, the obtainment of biomarkers during swallowing or meal consumption. Also, research on cough sounds in the framework of dysphagia is infrequent and has not reported conclusive results yet. Therefore, innovative methods allowing for the detection of cough reflex features in a natural setting (eg, bedside) could provide additional information regarding RAD.

## 7 CONCLUSIONS

Studies focusing on objective cough features are scarce, particularly in HNC‐patients with RAD. These patients may suffer from sensory deterioration, including an ineffective cough reflex, up to several years after treatment. This may lead to silent aspiration‐related pneumonia that can be fatal for a significant number of patients. Automatic or semi‐automatic cough sound analysis is a growing line of research that has provided valid measurements, particularly for assessing respiratory diseases. Despite scarce literature regarding cough features in the framework of dysphagia, exploring cough sounds might represent an alternative assessment method in HNC‐patients with RAD. Such a method is easily implementable in daily life and could provide novel information regarding these patients. To our knowledge, voluntary and reflexive acoustic cough features in HNC‐patients with RAD have never been explored. Hence, research focusing on acoustic cough‐related features should be considered for tracking dysphagia and/or aspiration in HNC‐patients with RAD.

## CONFLICT OF INTEREST

The authors declare that they have no competing interests.

## AUTHORS' CONTRIBUTIONS

All authors had full access to the data in the study and take responsibility for the integrity of the data and the accuracy of the data analysis. *Conceptualization*, S.M.B., G.V.N., J.S., M.D.B., D.V.G.; *Methodology*, F.M.L.; *Writing ‐ Original Draft*, S.M.B., G.V.N., J.S., D.V.G.; *Writing ‐ Review & Editing*, S.M.B., G.V.N., J.S., M.D.B., T.D., A.D., N.R., D.V.G.; *Supervision*, G.V.N., J.S., D.V.G., M.D.B.

## ETHICAL STATEMENT

Not applicable.

## Data Availability

Data sharing is not applicable to this article as no datasets were generated or analyzed during the current study.
